# New Appliances and Things Medical

**Published:** 1897-02-27

**Authors:** 


### NEW APPLIANCES AND THINGS MEDICAL.

**[We shall be glad to receive, at our Office, 28 & 29, Southampton Street, Strand, London, W.C., from the manufacturers, specimens of all new preparations and appliances which may be brought out from time to time.]**

### CYTOS BREAD.

(VICTORIA AND HAVEN ROLLER FLOUR MILLS, GRIMSBY.)

We have recently received from the above firm a specimen of their "Cytos" bread. It is partially malted and in a large measure manufactured from the germ of wheat. Two important results follow from the observance of the above conditions: (1) The nutritive value compares favourably with ordinary bread; (2) it is more easily digested and absorbed. Chemical analysis shows that the flour from which "Cytos" bread is manufactured contains a high percentage of albuminoid principles, and, in fact, in proportion to the carbohydrate content, is in correct value for the maintenance of life without any other proteid whatsoever. The carbohydrates as they exist in Cytos bread are partially soluble and partially insoluble, but the proportion of soluble carbohydrates is unusually high in comparison with that in other breads of a similar nature which we have had an opportunity of examining. From a consideration of the thoroughly scientific method of manufacture, from the pleasantness of flavour, from its composition and the ready manner in which it is digested and absorbed, we do not hesitate to recommend it to the notice of those who require in their bread the fulfilment of these conditions.

### NON-FARINACEOUS INFANTS' FOOD.

(CALLARD AND CO., 65, REGENT STREET, W.)

This new food for infants recommends itself to us on several grounds. In the first place the very name should be of service to the succeeding generation by the suggestiveness of the title, laying stress as it does on the necessity for feeding children on foods free from farinaceous elements or unconverted starch. Not one mother in twenty realises the importance of this fundamental law. Not only amongst the upper classes but amongst the poorest of the poor it is daily becoming a matter of greater necessity to resort to artificial methods of infant feeding. Callard's infants' food when prepared according to the directions given, that is to say, one table-spoonful added to half-a-pint of milk and half-a-pint of water, constitutes, in a physiological sense, a "perfect" food. It is easily digested, contains sufficient fatty and albuminoid elements, and what is equally important renders the milk, to which it is added, more digestible. Ordinary milk or milk and water forms in the stomach a heavy, bulky clot, very irritating to certain infant stomachs. The clot after the addition of Callard's food is fine, flocculent, easily digested, and free from irritating properties.

### NEW EAR CHANNEL.

(REYNOLDS AND BRANSON, BRIGGATE, LEEDS.)

The above firm have introduced one or two useful innovations in the apparatus used in aural practice, and the latest of these is a new ear channel. It has the advantage over the older varieties in dispensing with troublesome head springs, elastic loops, &c. It is readily maintained in position by a trigger which is held by a pin at one end, and can be brought down over the top of the ear and thus keeps the channel in position close to the head, no matter what be the size of the auricle. The basin is of sufficient size to catch all the outflowing water.

### ASEPTIC MEMBRANE PERFORATOR.

(REYNOLDS AND BRANSON, BRIGGATE, LEEDS.)

The new perforator, which is to be used in the rupture of the fœtal membranes, is intended to replace the homely but unscientific hair pin or finger-nail. It has been designed by Herbert Ribson, and it is made by Reynolds and Branson in celluloid, glass, or vulcanite. It is kept aseptic in a little glass bottle containing solution of perchloride of mercury. It is small and handy and makes no material addition to the usual obstetric impedimenta. It is about three inches long and in the form of a sharp-pointed flexible probe.

